# Synchronous telehealth and face‐to‐face administration of the Alberta Infant Motor Scale

**DOI:** 10.1111/dmcn.16391

**Published:** 2025-06-27

**Authors:** Kate L. Rawnsley, Jeanie L. Y. Cheong, Katharine Bennett, Melinda L. Mahady, Suzanne E. Smith, Diana Zannino, Louisa Remedios, Alicia J. Spittle

**Affiliations:** ^1^ Clinical Sciences Murdoch Children's Research Institute Melbourne Australia; ^2^ Department of Physiotherapy University of Melbourne Melbourne Australia; ^3^ Newborn Research Royal Women's Hospital Melbourne Australia; ^4^ Department of Obstetrics, Gynaecology and Newborn Health University of Melbourne Melbourne Australia; ^5^ Department of Paediatrics University of Melbourne Melbourne Australia; ^6^ Department of Physiotherapy Joan Kirner Women's and Children's Hospital Melbourne Australia; ^7^ Clinical Epidemiology and Biostatistics Unit Murdoch Children's Research Institute Melbourne Australia; ^8^ Institute of Health and Wellbeing Federation University Churchill Australia

## Abstract

**Aim:**

To determine the agreement between the Alberta Infant Motor Scale (AIMS), when delivered via synchronous telehealth compared with face‐to‐face administration to assess gross motor development of infants.

**Method:**

In this prospective cross‐sectional study, 123 infants (gestational age: mean 38.8 weeks (SD 1.8); range 31–42 weeks; male *n* = 65) were assessed at 4 months, 8 months, or 12 months old with two AIMS assessments: face‐to‐face and via synchronous telehealth. The agreement between the assessments was examined using intraclass correlation coefficient (ICC) and the Bland–Altman method with 95% limits of agreement.

**Results:**

Agreement between AIMS assessments administered face‐to‐face and via synchronous telehealth had an overall ICC of 0.99 (95% confidence interval [CI] 0.98, 0.99) and within age group: 4 months ICC 0.72 (95% CI 0.58, 0.83), 8 months ICC 0.97 (95% CI 0.96, 0.98), and 12 months ICC 0.98 (95% CI 0.96, 0.99).

**Interpretation:**

The AIMS assessment delivered via synchronous telehealth shows excellent agreement with face‐to‐face assessment. Telehealth is a good alternative to face‐to‐face AIMS assessment, particularly for older infants.

## Graphical abstract

The Alberta Infant Motor Scale (AIMS) is a gross motor assessment with excellent psychometric properties and clinical utility when used face‐to‐face. The AIMS assessment delivered via synchronous telehealth shows excellent agreement with face‐to‐face assessment. Telehealth is a suitable alternative to face‐to‐face AIMS assessment, particularly for older infants.

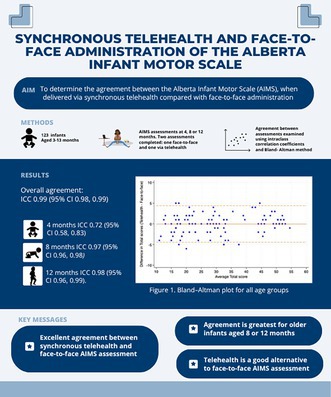

Plain language summary: https://onlinelibrary.wiley.com/doi/10.1111/dmcn.16448

**Abbreviations**
AIMS: Alberta Infant Motor Scale
ICC: intraclass correlation coefficient

**What this paper adds**

- Agreement between synchronous telehealth and face‐to‐face Alberta Infant Motor Scale assessments is excellent.
- Agreement is greatest for older infants aged 8 months or 12 months.

Gross motor assessments are an important component of paediatric developmental evaluation.^1^ These assessments may be used to detect developmental delay, monitor a child's progress over time, or evaluate the child's response to an intervention. The Alberta Infant Motor Scale (AIMS) is an observational assessment for gross motor development of children from birth until independent walking, widely used by health professionals.^1^ When administered face‐to‐face, the AIMS has excellent psychometric properties and clinical utility^1^, ^2^, ^3^ making it a commonly used tool internationally in both clinical and research settings.^1^

During the global COVID‐19 pandemic there was an increased reliance on telehealth for early developmental monitoring.^4^ Telehealth is a form of digital health which uses information and communication technologies to deliver health care.^5^ This rapid shift in health care delivery highlighted the sparsity of standardized developmental assessments validated for use via telehealth.^6^, ^7^, ^8^ Beyond the pandemic, telehealth has the potential to improve access to skilled clinicians for families, regardless of geographic location.^9^ In addition, telehealth may reduce the burden associated with attending health care appointments, eliminate travel time, reduce costs, and build family capacity.^10^ However, for telehealth to be successful and sustainable, valid assessment tools are needed for early detection and monitoring of developmental delay.

A recent scoping review explored the psychometric properties and feasibility of the AIMS used via telehealth.^11^ Although evidence was limited, authors suggested the AIMS showed potential for successful use via telehealth. While agreement between synchronous telehealth (i.e. live video consultation) and face‐to‐face administration of the English version of the AIMS has not been established, there have been studies investigating the agreement of synchronous use of the Brazilian‐Portuguese version of the AIMS^12^ and another with asynchronous (i.e. prerecorded) AIMS assessment, whereby the English version of the assessment was adapted to facilitate filming at home by parents.^13^ Both studies showed encouraging results but had their limitations. Given the limited cross‐cultural validity of any standardized motor developmental assessment because of differences in the environment, sociocultural beliefs, and caregiving practices,^14^, ^15^ the use of the Brazilian version of the AIMS limits the study's generalizability. Prerecorded videos also have clinical barriers such as capability of families to self‐record, technology requirements to securely upload videos, and reimbursement of clinicians' time to evaluate the video.^16^ The aim of this study was to determine the agreement between the English version of the AIMS assessment when delivered via synchronous telehealth, where the caregiver is guided by a physiotherapist, compared with face‐to‐face AIMS assessment.

## METHOD

### Participants

In this prospective cross‐sectional study, participants were recruited from Metropolitan Melbourne and the regional town of Bendigo, Victoria, Australia between May 2023 and March 2024. These locations were selected to allow further research into the perspectives of telehealth use of those living in metropolitan and regional areas. Prospective recruitment was primarily through social media along with Bendigo maternal and child health nurses and new parent groups through snowball sampling. A target sample size of a minimum 120 infants was set based on an acceptable intraclass correlation coefficient (ICC) of no less than 0.8 and a 95% confidence interval (CI) width of 0.1. Infants born at term or preterm were included from birth to 12 months of age, with assessments scheduled at, or within 2 weeks of, 4 months, 8 months, or 12 months of age (corrected age for infants born preterm). These age groups were selected to broadly reflect the different developmental stages of infancy, allowing age‐dependent effects on agreement to be investigated. Infants were excluded if they were older than 13 months at time of enrolment, were walking independently, were in out‐of‐home care, or resided outside of approximately a 25 km radius from the Royal Women's Hospital or centre of Bendigo. Ethics approval was obtained from the Human Research Ethics Committees at the Royal Women's Hospital (HREC/87540/RWH 22–26). Parents provided written informed consent (electronically) to participate. Study design was influenced by the lived experience of author KLR who is the parent of a medically complex child, living regionally. KLR has received telehealth services as a parent as well as provided telehealth services to clients as a health professional.

### Assessments

Basic medical and demographic information was obtained from the child's caregiver using an online REDCap (electronic data capture tool) survey before the first assessment. Assessors were blinded to this information with the exception of birthdate and gestational age as these are required to complete the assessment ranking. Information collected included gestational age at birth, birth or neonatal complications, level of maternal education, and the primary language spoken at home (English only vs. other). A home visit safety screening was completed over the phone before the appointment.

Assessments were completed by any one of four paediatric physiotherapists, experienced in completing the AIMS and with at least 8 years of paediatric physiotherapy experience. A training day was held before data collection commencing and raters concurrently scored four infants. Raters independently scored the infants and then scores were compared and discussed until absolute agreement with expert rater (AJS; associate editor of the AIMS Second Edition, KLR) was reached.

The face‐to‐face AIMS assessments were completed at the participants' home. Sessions were recorded and uploaded to a secure University of Melbourne server. Within 2 weeks, either before or after the face‐to‐face session, the AIMS assessment was completed via telehealth with the same participant. Families were sent a telehealth set‐up guide and video conference link via email beforehand. The assessor guided the caregiver through the assessment, using a doll to demonstrate. To remove possible bias from the family being familiar with the assessing clinician and to allow clinicians to be blinded to the other assessment results, a different clinician completed each assessment. The order of the assessments (i.e. face‐to‐face or telehealth first) was randomized to reduce bias from parents being familiar with the assessment. Randomization occurred after families consented to the study using REDCap and were stratified based on location (i.e. metropolitan vs. regional) and age of child.

Families were given general feedback at the end of the telehealth assessment; however, AIMS scores and centiles were provided to the families after the face‐to‐face assessments only, as this is the setting in which the scale has been validated. If developmental concerns were apparent, the assessor advised the family to seek a private physiotherapist or speak to their general practitioner or maternal and child health nurse to request referral to community services and a letter was provided. If an infant had a score difference greater than 2 points between assessments, the video recording was reviewed to understand where variations were occurring.

As part of a larger study, families were sent an electronic survey after completion of both assessments asking parents about their AIMS experiences. A purposive maximum variation sample^17^ of parents was selected to participate in semi‐structured interviews. Findings of the surveys and interviews are beyond the scope of this paper and will be published separately.

### Statistical analyses

Data were analysed using Stata version 18 (StatCorp, College Station, TX, USA). Participant characteristics were summarized as mean (SD) or proportions. The agreement between the two AIMS assessments (telehealth vs. face‐to‐face) were examined using ICC calculated using linear mixed models, with cluster‐robust standard errors (to account for clustered observations) where the AIMS delivery method was the fixed effect and the participant was the random effect. The Bland–Altman method^18^ with 95% limits of agreement was also used. Interpretation of the ICCs were based on the cut‐offs:^17^ more than 0.90 excellent reliability; 0.75 to 0.9 good reliability; 0.5 to 0.75 moderate reliability; and less than 0.50 poor reliability. A linear mixed model accounting for the clustered observations was used to assess the effect of the randomization ordering on the difference between the two AIMS assessments. Analyses were performed for the full cohort as well as separately for each age subgroup. Absolute agreement, reported as a percentage, was achieved if the difference between telehealth and face‐to‐face assessments was zero.

## RESULTS

One hundred and twenty‐six infants were recruited from metropolitan (*n* = 80; 63.5%) and regional (*n* = 46; 36.5%) locations. Of the 126 infants recruited, 123 participated in AIMS assessments (male *n* = 65; metropolitan *n* = 78; regional *n* = 45). There were three participant withdrawals because of scheduling difficulties before either assessment was completed. Participants were divided into 4‐month (*n* = 48), 8‐month (*n* = 50), or 12‐month (*n* = 25) cohorts with assessments occurring within 2 weeks of the allocated age (assessment age range: 3.75–13.5 months). Gestational age at birth ranged from 31 to 42 completed weeks. Participant characteristics are further described in Table 1 and participant flow is shown in Figure S1. In one case, a face‐to‐face assessment score was reduced by 1 point after reviewing the video recording. This was because of a parent providing subtle assistance while the child was pulling to stand that was not visible to the live assessor.

**TABLE 1:** Characteristics of participants (*n* = 123).

Participant characteristics	Summary
Gestational age (completed weeks)	38.8 (1.8)
Birthweight (g)	3373.1 (621.9)
Male	65/123 (52.9%)
Multiple pregnancy	10/123 (8.1%)
Postsecondary education/training	120/123 (97.6%)
Only English spoken at home	108/123 (87.8%)
NICU/SCN admission	20/123 (16.3%)
Low birthweight <2500 g	12/123 (9.8%)
Preterm <37 weeks GA	14/123 (11.4%)

Data are presented as mean (SD) for continuous measures, and *n*/total responders (%) for categorical measures.

Abbreviations: GA, gestational age; NICU, neonatal intensive care unit; SCN, special care nursery.

### All age groups

**FIGURE 1:**
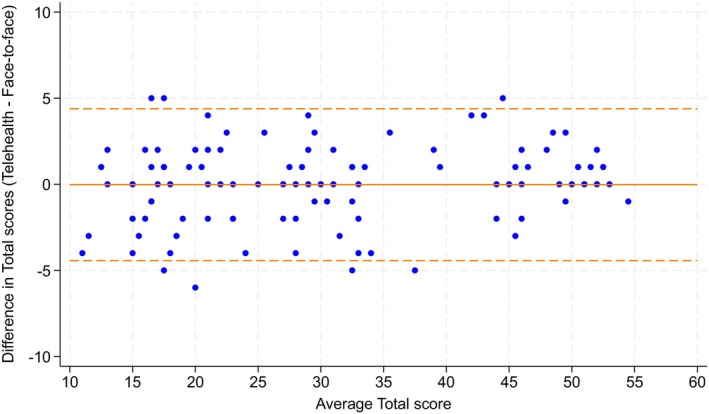
Bland–Altman plots for all age groups.

The overall agreement between AIMS assessments administered face‐to‐face and via synchronous telehealth was excellent with an ICC of 0.99 (95% CI 0.98, 0.99). The average difference between assessment scores was −0.24 (SD 2.23). Bland–Altman analysis showed 95% limits of agreement of −4.39, 4.34. Results of Bland–Altman analyses are illustrated in Figure 1. There were 93 infants (75.6%) that had a difference between scores of 2 or less items with 36 of these (29.3%) having absolute agreement. There was negligible difference in overall agreement (ICC 0.99, 95% CI 0.98, 0.99) when analysis was confined to the 103 infants who received both assessments within 1 week. Agreement for individual assessment items is shown in Table S1.

### The 4‐month‐old cohort

**FIGURE 2:**
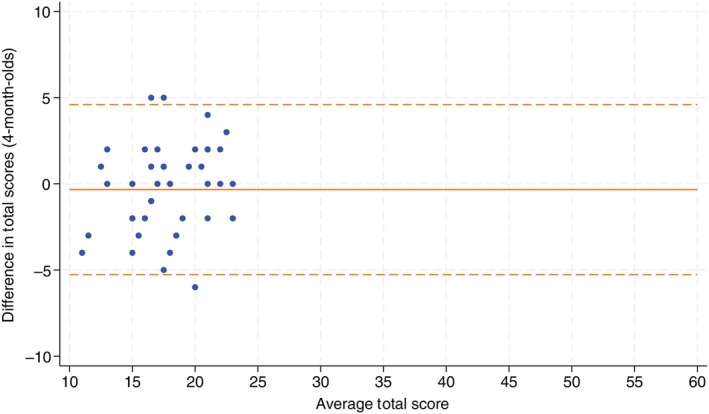
Bland–Altman plots for 4‐month‐old cohort.

Forty‐eight infants were assessed in the 4‐month‐old cohort. Agreement was moderate with an ICC of 0.72 (95% CI 0.58, 0.83). The average difference between assessment scores was −0.33 (SD 2.45) with 95% limits of agreement of −5.14, 4.47. Figure 2 shows the Bland–Altman plot for the 4‐month‐old cohort. For 4‐month‐old infants specifically, there was a marked reduction in agreement when assessments were completed more than 1 week (but <2 weeks) apart with an ICC of 0.35 (95% CI 0.02, 0.94). When assessments were both completed within 1 week, agreement was good with an ICC of 0.75 (95% CI 0.61, 0.85). Given the lower agreement in the 4‐month‐old age group, an ad hoc analysis of agreement for the individual assessment items was performed and is shown in Table S1.

### The 8‐month‐old cohort

**FIGURE 3:**
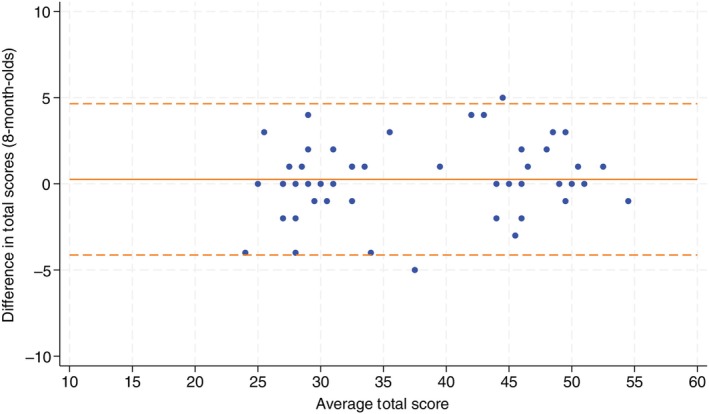
Bland–Altman plots for 8‐month‐old cohort.

Fifty infants were assessed in the 8‐month‐old cohort. Agreement was excellent with an ICC of 0.97 (95% CI 0.96, 0.98). When analysis was confined to the 42 infants who had both assessments within 1 week, the ICC was 0.98 (95% CI 0.96, 0.99). As Figure 3 shows, the average difference between face‐to‐face and telehealth assessments was 0.26 (SD 2.18) with 95% limits of agreement of −4.02, 4.54.

### The 12‐month‐old cohort

**FIGURE 4:**
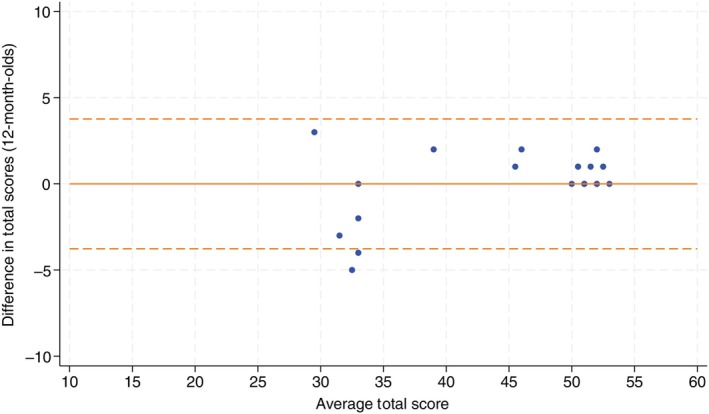
Bland–Altman plots for 12‐month‐old cohort.

Twenty‐five infants were assessed in the 12‐month‐old cohort. Agreement was excellent with an ICC of 0.98 (95% CI 0.96, 0.99). There was negligible difference when analysis was confined to the 21 infants who completed both assessments within 1 week: ICC 0.98 (95% CI 0.95, 0.99). On average there was no difference between face‐to‐face and telehealth assessment scores: 0.00 (SD 1.83). The 95% limits of agreements based on Bland–Altman analysis are −3.58, 3.58 (Figure 4).

### Assessment order

While there was evidence of an effect depending on assessment order, the size of the difference was small. When the AIMS was assessed face‐to‐face, infants who had the face‐to‐face assessment first, on average scored 0.85 (95% CI –4.05, 5.76) items higher than those who had the telehealth assessment first. When the AIMS was assessed via telehealth, infants who had had the face‐to‐face assessment first on average scored 1.55 (95% CI 0.83, 2.26) items higher than those who had the telehealth assessment first. Regardless of assessment order, on average, there was little difference between assessments with infants scoring better on their second assessment by 0.06 items (95% CI –0.65, 0.77). Overall, the difference between delivery methods was negligible with face‐to‐face assessment scores being 0.02 (95% limits of agreement −4.34, 4.39) items better than telehealth scores.

## DISCUSSION

In this Australian‐based cohort of infants spanning a broad range of gestational ages at birth, there was excellent agreement between AIMS assessments delivered via synchronous telehealth compared with face‐to‐face administration. Agreement was greatest for older infants (8‐month‐old and 12‐month‐old cohorts) and lower for 4‐month‐olds. This is consistent with findings from the AIMS face‐to‐face reliability study where differences between assessment scores were largest for infants aged 4 to 7 months old.^1^ Telehealth offers a suitable alternative to face‐to‐face AIMS assessment in this population, particularly for older infants.

Given the nature of the study, it is difficult to ascertain the precise reason for score differences. The influence of interrater reliability, infant state, time, and true developmental changes in rapidly developing infants is difficult to differentiate from assessment method differences. In a reliability study of face‐to‐face AIMS administration on two occasions up to 1 week apart scored by different assessors, the standard error of measurement was equivalent to two items.^1^ In our study, just over 75% (93/123) of infants had assessment score differences of 2 or less items. In many cases where score differences were greater than 2, assessors had noted unfavourable infant states such as being tired or irritable or that parents reported the infant had gained new skills since the previous assessment. In the 4‐month cohort, agreement was poor when assessments were completed more than 1 week apart. It is likely these differences were due to rapidly changing motor skills at this age, not the assessment delivery method. The overall lower agreement for the 4‐month cohort may be due to the less predictable routines at this age making scheduling for calm, awake windows more difficult. Further, the items frequently observed at this age require more facilitation or encouragement (e.g. sitting with propped arms, pull to sit, rolling). During the telehealth sessions, the parent was responsible for facilitating these items. The 1.5 item increase in telehealth scores if an infant had already been assessed face‐to‐face may suggest a small learning effect (i.e. parents may have been better at facilitating movements if they had previously seen a physiotherapist do the assessment). Clinically though, this difference is unlikely to be meaningful.

Our results are consistent with previous findings. A recent Brazilian study assessed 77 infants face‐to‐face and via synchronous telehealth using the Brazilian version of the AIMS^12^ while a Netherlands‐based study compared face‐to‐face AIMS assessments with prerecorded videos of 48 term‐born infants.^13^ Both studies reported an ICC of 0.99 and a mean difference between scores of 0.60 and 0.46 respectively. In the Brazilian cohort, 80% of the infants assessed had a difference between scores of 2 or less items.^12^ Both studies reported parents were accepting of the telehealth assessment. A published commentary regarding the Netherlands‐based asynchronous study^16^ has highlighted limitations of using prerecorded home videos for the AIMS, including potential inability of some families to perform the assessment, and limited service models that sufficiently fund therapist evaluation of video assessments.^16^ Parents who participated in the asynchronous study also reported barriers with time planning the videos and technical difficulties uploading the recordings.^19^ While synchronous telehealth delivery addresses many of these barriers, it has its own limitations such as billing models for telehealth services and reduced flexibility in assessment scheduling compared to asynchronous methods. Despite these challenges, both synchronous and asynchronous telehealth have the potential to improve access for families living regionally or remotely. Recording synchronous telehealth sessions or using prerecorded videos also has the potential to improve reliability, allowing for later viewing or discussion with a second rater.

The study design, including sample size, randomized assessment order, blinding of assessors to previous assessment results, and inclusion of both metropolitan and regional families, was a strength of this study. The time interval between assessments is a limitation. The true agreement between telehealth and face‐to‐face AIMS if assessed simultaneously may be higher than our reported findings. Separate assessment appointments were chosen in an attempt to mimic the clinical scenario for the remote assessment, whereby the caregiver and child are in their own environment without an unfamiliar person. While the second assessment was scheduled as soon as possible after the first, a 2‐week interval was allowed for pragmatic reasons. Given the excellent overall agreement reported in this study, while reducing the time interval may further improve agreement, it is unlikely to affect the clinical recommendations.

A further limitation is the effects of interrater reliability on score differences. This was minimized by all assessors being experienced paediatric physiotherapists, confident in administering the AIMS and providing opportunities for co‐scoring to ensure agreement before commencing the study. For infants with larger score differences (>2 points), the assessment video recordings were reviewed to ensure score accuracy.

The majority of families (87.8%) spoke only English at home and most primary caregivers (96.8%) had postsecondary education or training. This is likely due to recruitment material being written in English and caregivers were required to initiate contact electronically to express interest in the study. Naturally this recruitment method attracted an educated, computer‐literate sample. Thus, our results may not be generalizable to the broader community. Similarly, the Netherlands‐based study reported a highly educated sample of caregivers with almost 75% having advanced education.^13^ Future research should consider targeted recruitment of non‐English‐speaking and socially vulnerable or low‐resourced families. The original English version of the AIMS using Canadian‐based norms was used for this English‐speaking cohort. Consideration of cultural adaptation of the AIMS in countries outside of the norm‐referenced population, regarding context‐specific factors, has previously been recommended, even in native English‐speaking countries.^15^ As this study compared raw scores of the two assessments, cultural implications are unlikely to have affected agreement; however, they are a consideration for the administration and interpretation of the assessment itself.

The COVID‐19 pandemic and associated restrictions in face‐to‐face health care have seen a marked increase in telehealth‐related research in recent years. The overlap in timing of our study with the Brazilian‐based cohort highlights the global interest in establishing valid, telehealth‐based assessment measures. The similar findings across both studies highlight the potential for broad clinical use internationally, in both high‐income and middle‐income countries. The AIMS assessment delivered via telehealth shows excellent agreement with face‐to‐face assessment and should be considered as part of clinical care, particularly for older infants.

Telehealth sessions took approximately 20 minutes to complete whereas face‐to‐face sessions, when accounting for travel, generally required 75 to 90 minutes of clinician time. Telehealth assessment may therefore offer a time‐effective alternative to home‐based assessments, while still allowing the infant to be viewed in their natural environment. In general, telehealth has been reported to be well‐accepted by families;^6^, ^7^, ^12^, ^13^ however, further research into the perspectives of families and suggestions to optimize the accessibility and acceptability of AIMS assessments via telehealth would be beneficial but is beyond the scope of this paper.

The AIMS assessment delivered via synchronous telehealth, where the caregiver is guided by a health professional, is a suitable alternative to face‐to‐face AIMS administration for infants. Telehealth offers an effective, time‐efficient assessment option for clinical or research purposes. Family preferences, access, and confidence with technology should be considered when selecting a face‐to‐face or telehealth‐based AIMS assessment.

## CONFLICT OF INTEREST STATEMENT

AJS is associate editor of the AIMS manual (*Motor Assessment of the Developing Infant*).

## Supporting information


**Figure S1:** Participant flow.

**Table S1:** Agreement for individual AIMS items.

## Data Availability

The data that support the findings of this study are available from the corresponding author upon reasonable request.

## References

[1] Piper M , Darrah J . Motor Assessment of the Developing Infant. 2^nd^ ed. St. Louis: Elsevier Health Sciences; 2022.

[2] Eliks M , Gajewska E . The Alberta Infant Motor Scale: A tool for the assessment of motor aspects of neurodevelopment in infancy and early childhood. Frontiers in neurology. 2022;13:927502. 36188401 10.3389/fneur.2022.927502 PMC9515325

[3] Spittle AJ , Doyle LW , Boyd RN . A systematic review of the clinimetric properties of neuromotor assessments for preterm infants during the first year of life. Developmental Medicine & Child Neurology. 2008;50(4):254–66. 18190538 10.1111/j.1469-8749.2008.02025.x

[4] Srinivasan R , Wallis KE , Soares N . Global trends in telehealth among clinicians in developmental‐behavioral pediatric practice: a COVID‐19 snapshot. Journal of Developmental & Behavioral Pediatrics. 2022;43(1):32–7. 33990511 10.1097/DBP.0000000000000963

[5] The International Organisation for Standardisation . Health informatics‐ telehealth services [internet]. 2014 [Available from: https://www.iso.org/obp/ui/#iso:std:iso:ts:13131:ed‐1:v1:en.

[6] La Valle C , Johnston E , Tager‐Flusberg H . A systematic review of the use of telehealth to facilitate a diagnosis for children with developmental concerns. Res Dev Disabil. 2022;127:104269. 35636261 10.1016/j.ridd.2022.104269 PMC10521149

[7] Zischke C , Simas V , Hing W , Milne N , Spittle A , Pope R . The utility of physiotherapy assessments delivered by telehealth: A systematic review. Journal of global health. 2021;11. 10.7189/jogh.11.04072 PMC8684795 34956637

[8] DeMauro SB , Duncan AF , Hurt H . Telemedicine use in neonatal follow‐up programs – What can we do and what we can't – Lessons learned from COVID‐19. Seminars in Perinatology. 2021;45(5):151430. 33892961 10.1016/j.semperi.2021.151430 PMC8022519

[9] World Health Organisation . Recommendations on Digital Interventions for Health System Strengthening: Evidence and recommendations [Internet].2019 February 1, 2021. 31162915

[10] Early Childhood Intervention Australia . Telepractice for early childhood intervention practitioners [internet]. 2020.[Available from https://www.flipsnack.com/earlychildhoodintervention/ecia‐telepractice‐for‐eci‐practitioners‐april‐2020/full‐view.html]

[11] Passamani RS , de Vargas Ciello H , Brugnaro BH , Dos Santos AN . The psychometric properties and feasibility of the Alberta infant motor scale used in telehealth: A scoping review. Early Human Development. 2024:105941. 38237305 10.1016/j.earlhumdev.2024.105941

[12] Passamani RS , Shigihara CK , Gomes PG , dos Santos AN . Agreement of synchronous remote and in‐person application of the Alberta Infant Motor Scale: Cohort study. Journal of Telemedicine and Telecare. 2024;0(0):1357633X241245160. 10.1177/1357633X241245160 38659374

[13] Boonzaaijer M , van Dam E , van Haastert IC , Nuysink J . Concurrent Validity Between Live and Home Video Observations Using the Alberta Infant Motor Scale. Pediatric physical therapy: the official publication of the Section on Pediatrics of the American Physical Therapy Association. 2017;29(2):146–51. 10.1097/PEP.0000000000000363 PMC5374751 28350771

[14] Mendonça B , Sargent B , Fetters L . Cross‐cultural validity of standardized motor development screening and assessment tools: A systematic review. Developmental Medicine & Child Neurology. 2016;58(12):1213–22. 27699768 10.1111/dmcn.13263

[15] Mendonça B , Kong M , Coombs A , Kysh L , Sargent B . Psychometric properties of the Alberta Infant Motor Scale and culturally adapted or translated versions when used for infant populations internationally: A systematic review. Dev Med Child Neurol. 2025;67(2):165–76. 39234875 10.1111/dmcn.16070 PMC11695772

[16] Gatlin RB , Hausch S , Caruso L . Commentary on ‘Concurrent Validity Between Live and Home Video Observations Using the Alberta Infant Motor Scale’. Pediatric physical therapy: the official publication of the Section on Pediatrics of the American Physical Therapy Association. 2017;29(2):152. 10.1097/PEP.0000000000000385 28350772

[17] Etikan I. Comparison of Convenience Sampling and Purposive Sampling. American Journal of Theoretical and Applied Statistics. 2016;5:1.

[18] Bland J. Statistical methods for assessing agreement between two methods of clinical measurement. Lancet. 1986. 2868172

[19] Boonzaaijer M , van Wesel F , Nuysink J , Volman MJM , Jongmans MJ . A home‐video method to assess infant gross motor development: parent perspectives on feasibility. BMC pediatrics. 2019;19(1):392‐. 31664955 10.1186/s12887-019-1779-x PMC6819354

