# Supplementary figures and images for: Synchronous telehealth and face‐to‐face administration of the Alberta Infant Motor Scale
Source: Dev Med Child Neurol. 2025 Jun 27;68(1):91–8. doi: 10.1111/dmcn.16391 (PMC12683301; doi:10.1111/dmcn.16391)

**Figure S1. Participant Flow**

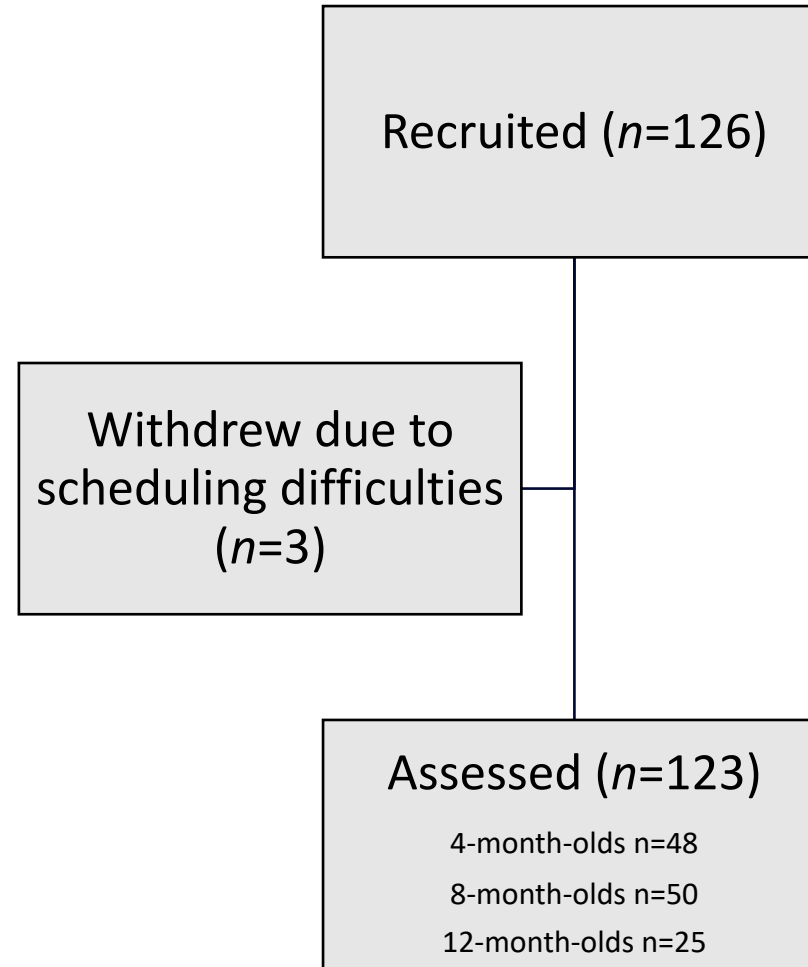

Supplement: Supplementary file 1 — Figure S1: Participant flow. [file DMCN-68-91-s001.pdf]
