# Supplementary material for: Synchronous telehealth and face‐to‐face administration of the Alberta Infant Motor Scale
Source: Dev Med Child Neurol. 2025 Jun 27;68(1):91–8. doi: 10.1111/dmcn.16391 (PMC12683301; doi:10.1111/dmcn.16391)
Supplement: Supplementary file 2 — Table S1: Agreement for individual AIMS items. [file DMCN-68-91-s002.docx]

**Table S1. Agreement (%)* for individual AIMS items**

| Item | Agreement (%)  All ages | Agreement (%)  4-month-olds |
| --- | --- | --- |
| Prone Lying (1) | - | - |
| Prone Lying (2) | - | - |
| Prone Prop | 99.2 | 97.9 |
| Forearm Support (1) | 98.4 | 95.8 |
| Prone Mobility | 96.8 | 91.7 |
| Forearm Support (2) | 95.1 | 89.6 |
| Extended Arm Support | 84.6 | 68.8 |
| Rolling Prone to Supine Without Rotation | 84.6 | 81.3 |
| Swimming | 87.0 | 87.5 |
| Reaching From Forearm Support | 84.6 | 72.9 |
| Pivoting | 86.2 | 79.2 |
| Rolling Prone to Supine With Rotation | 88.6 | 95.8 |
| Four-Point Kneeling (1) | 91.9 | - |
| Propped Sidelying | 92.7 | - |
| Reciprocal Crawling | 96.8 | - |
| Four-Point Kneeling to Sitting or Half-sitting | 94.3 | - |
| Reciprocal Creeping (1) | 92.7 | - |
| Reaching from Extended Arm Support | 92.7 | - |
| Four-Point kneeling (2) | 95.9 | - |
| Modified Four-Point Kneeling | 96.8 | - |
| Reciprocal Creeping | 95.1 | - |
| Supine Lying (1) | - | - |
| Supine Lying (2) | - | - |
| Supine Lying (3) | - | - |
| Supine Lying (4) | 99.2 | 97.9 |
| Hands to Knees | 89.4 | 79.2 |
| Active Extension | 77.2 | 62.5 |
| Hands to Feet | 90.2 | 85.4 |
| Rolling Supine to Prone Without Rotation | 87.7 | 85.1 |
| Rolling Supine to Prone With Rotation | 85.3 | 85.1 |
| Sitting With Support | - | - |
| Sitting With Propped Arms | 89.4 | 72.9 |
| Pull to Sit | 93.5 | 83.3 |
| Unsustained Sitting | 92.7 | 81.3 |
| Sitting With Arm Support | 98.4 | 95.8 |
| Unsustained Sitting Without Arm Support | 100.0 | - |
| Weight Shift in Unsustained Sitting | 100.0 | - |
| Sitting Without Arm Support (1) | 97.6 | - |
| Reach With Rotation in Sitting | 96.8 | - |
| Sitting to Prone | 91.1 | - |
| Sitting to Four-Point Kneeling | 95.1 | - |
| Sitting Without Arm Support (2) | 92.7 | - |
| Supported Standing (1) | - | - |
| Supported Standing (2) | 100.0 | 100.0 |
| Supported Standing (3) | 89.3 | 85.1 |
| Pulls to Stand With Support | 96.7 | - |
| Pulls to Stand/Stands | 95.9 | - |
| Supported Standing With Rotation | 94.2 | - |
| Cruising Without Rotation | 89.3 | - |
| Half-kneeling | 95.0 | - |
| Controlled Lowering Through Standing | 94.2 | - |
| Cruising With Rotation | 95.9 | - |
| Stands Alone | 97.5 | - |
| Early Stepping | 100.0 | - |
| Standing From Modified Squat | 100.0 | - |
| Standing From Quadruped Position | - | - |
| Walks Alone | - | - |
| Squat | 99.2 | - |

* Agreement = the percentage of concordant scoring

**‘-‘ = All infants scored in the same category therefore agreement was not scored

Note: Agreement for the 4-month-old cohort was analysed due to the lower overall ICC for this age group.
